# Association between histamine 2 receptor antagonists and sepsis outcomes in ICU patients: a retrospective analysis using the MIMI-IV database

**DOI:** 10.1186/s44158-023-00089-4

**Published:** 2023-02-09

**Authors:** Tarek R. Firzli, Sunil Sathappan, Daniel Antwi-Amoabeng, Bryce D. Beutler, Mark B. Ulanja, Farah Madhani-Lovely

**Affiliations:** 1 Reno School of Medicine, University of Nevada, 1664 N Virginia Street, Reno, NV 89557 USA; 2 Christus Ochsner St. Patrick Hospital, 524 Dr Michael Debakey Dr, Lake Charles, LA 70601 USA; 3 Keck School of Medicine, University of Southern California, 1500 San Pablo Street, 2nd Floor, Los Angeles, CA 90033 USA; 4 Department of Pulmonary and Critical Care Medicine, Renown Health, 1155 Mill St., Reno, NV 89502 USA

**Keywords:** Sepsis, Histamine antagonists, H2RA, MIMIC database, ICU, Mortality, Length of stay

## Abstract

**Background:**

Sepsis is marked by elevated histamine, which is a vasodilator that increases vascular permeability. Although human studies are lacking, murine models of sepsis have indicated potential protective effects of histamine 2 receptor antagonist administration (H2RAs).

**Objective:**

To assess any association between H2RA use in sepsis-3 patients admitted to the ICU and mortality, mechanical ventilation, length of stay, and markers of renal, liver, and lung dysfunction.

**Design:**

Retrospective cohort study.

**Setting:**

Intensive care units of the Beth Israel Deaconess Medical Center (BIDMC) accessed via the MIMIC-IV database spanning an 11-year period from 2008 to 2019.

**Patients (or participants):**

A total of 30,591 patients met the inclusion criteria for sepsis-3 on admission (mean age 66.49, standard deviation 15.92).

**Main measures:**

We collected patient age, gender, ethnicity, comorbidities (contained within the Charlson comorbidity index), SOFA score, OASIS score, APS III score, SAPS II score, H2RA use, creatinine, BUN, ALT, AST, and P/F ratios. Primary outcomes were mortality, mechanical ventilation, and ICU length of stay.

**Key results:**

A total of 30,591 patients met inclusion criteria over the 11-year sample period. The 28-day in hospital mortality rate was significantly lower among patients who received an H2RA (12.6% vs 15.1%, *p* < 0.001) as compared to those who did not receive an H2RA. Patients receiving an H2RA had significantly lower adjusted odds of mortality (0.802, 95% CI 0.741–0.869, *p* < 0.001), but significantly higher adjusted odds of invasive mechanical ventilation (4.426, 95% CI 4.132–4.741, *p* < 0.001) and significantly higher ICU LOS (3.2 days vs. 2.4 days, *p* < 0.001) as compared to the non-H2RA group. H2RA use was also associated with decreased severity of acute respiratory distress syndrome (ARDS) and lower serum creatinine.

**Conclusion:**

Among patients hospitalized in the ICU for sepsis, the use of an H2RA was associated with significantly lower odds of mortality, decreased severity of ARDS, and a lower incidence of renal insufficiency.

## Introduction

Among hospitalized patients, sepsis is associated with increased morbidity and mortality, prolonged length of stay (LOS), and higher healthcare costs. In addition, sepsis represents a leading cause of neurological and functional disability [1, 2]. Early recognition of sepsis and intervention with source control, antibiotics, and hemodynamic and ventilation optimization is critical to improve outcomes [3–5]. Adjunctive therapies for sepsis sometimes include corticosteroids [6] and experimental therapeutics such as liposomal agents, antibacterial antibodies, alkaline phosphatase, and interleukin-7 are currently under investigation [7].

Sepsis is associated with increased plasma concentration of histamine, which has vasodilatory effects at the capillary level [8, 9]. In mouse models of sepsis, activation of histamine-1 and 2 receptors contributed to the development of major organ damage deemed to be due to higher levels of proinflammatory cytokines with associated capillary rupture and vascular leak and resultant parenchymal lung damage, marked elevation in liver enzymes and BUN/creatinine levels [10]. In that study, the administration of intravenous famotidine (a histamine-2 receptor blocker) resulted in reduced incidence end organ damage [10], suggesting that histamine-2 receptors are involved in sepsis-related lung, liver, and kidney injuries. In fact, glucocorticoids are used even in the absence of adrenal insufficiency or shock to counteract the vasodilatory effects of histamine release in septic shock [6, 11].

However, there are limited data describing the relationship between histamine receptor antagonism and sepsis outcomes. The Surviving Sepsis Campaign recommends stress ulcer prophylaxis in patients with bleeding risk factors [12]. H2RAs and proton pump inhibitors (PPIs) are the most commonly used drugs for stress ulcer prophylaxis (SUP). Guidelines suggest that in critically ill patients, not exclusively sepsis patients, PPIs are preferred in patients with high risk of GI bleeding due to increased efficacy in decreasing clinically important bleeding (CIB) [13]. In patients with lower risk of CIB, there are no clear guidelines for use. H2RAs may also be preferred due to their lower cost, potential lower risk of pneumonia compared to proton pump inhibitors [14–18]. Furthermore, research has demonstrated that although CIB is decreased with PPIs compared to H2RAs, mortality has not been significantly different between groups, with mortality generally being lower with H2RA use [13, 19]. These studies all focus on patients who are critically ill, but not necessarily meeting sepsis-3 criteria. Due to the ubiquitous use of H2RAs both for treatment and prophylactic indications, potential mortality benefits of H2RAs, and early data suggesting histamine blockage could be beneficial in murine sepsis models, we sought to investigate the association between HR2A use and all-cause mortality, end organ damage, and hospital length of stay in ICU patients who specifically had sepsis by the current definition.

## Methods

### Summary of data source

We used data from the Medical Information Mart for Intensive Care IV (MIMIC-IV) database [20]. The MIMIC-IV is a publicly available, free database which contains a comprehensive catalog of individual patient-level information on hospital stays for patients admitted to a tertiary academic medical center in Boston, MA, USA and obtained from PhysioNet [21]. PhysioNet currently operates with funding from the National Institute of Biomedical Imaging and Bioengineering and offers data on several clinical parameters on over 40,000 individual subjects as well as tools to extract and analyze the data.

### Study population and outcomes

We queried the MIMIC-IV database for all adult intensive care unit (ICU) stays from 2008 to 2019. Patients 18 years or older, were included if they met the sepsis-3 criteria [22]. We acquired the following information for each patient indexed by stay ID: age, gender, and risk scores including APS III [23], SAPS II [24], SOFA [25], OASIS [26], and the Charlson comorbidity index [27]. We also extracted data on time from ICU admission to H2RA administration (if applicable), and time from ICU admission to invasive mechanical ventilation (IMV). We extracted ICU length of stay, number of days until in-hospital mortality, and in-hospital 28-day mortality. We assessed the severity of respiratory dysfunction with daily means of PaO2/FiO2 (PF ratio) over a 7-day period after admission for each patient. We further categorized the severity of pulmonary dysfunction based on the Berlin definition of acute respiratory distress syndrome, ARDS (severe ARDS: P/F ratio < 100 mmHg; moderate ARDS: P/F ratio < 200 mmHg; mild ARDS: P/F ratio < 300 mmHg) [28]. Additionally, kidney function and liver function were followed for each patient over the same 7-day period using daily means of BUN, creatinine, alanine aminotransferase (ALT), and aspartate aminotransferase (AST). Queries were performed in Google BigQuery [29]. Primary outcomes of interest included in-hospital mortality, ICU length of stay, and the use of IMV after day 1 of ICU stay. Secondary Outcomes included the mean P/F ratio, mean BUN/Cr ratio, AST, and ALT levels on days 1 through 7 of ICU stay. Patients were grouped by whether they had received H2RAs (ranitidine, famotidine, or cimetidine) from time of admission to 1 day of ICU stay (H2RA group) and those who did not (no H2RA group). We excluded patients who received H2RAs after day 1 of ICU admission as well as patients who were mechanically ventilated prior to receiving H2RAs. Patients with missing values in age, gender, comorbidities, or timestamps corresponding to H2RA use or mechanical ventilation (where these treatments were utilized) were also excluded.

### Statistical analyses

Continuous variables were summarized as means ± standard deviations or median (interquartile range), where appropriate. Categorical variables were summarized as counts (percentages). We assessed differences in 28-day mortality between groups using univariate analysis and used a multivariable logistic regression analysis including age, gender, risk scores, and comorbidities to examine between group differences. Utilizing the same patient groups as the mortality analysis, we evaluated the ICU length of stay between groups, first utilizing univariate analysis of length of stay. We then evaluated differences in a multivariable linear regression model including age, gender, risk scores, and comorbidities. Risk for IMV was assessed with univariate analysis as well as in multivariable logistic regression. Subgroup analysis of IMV and H2RA administration was performed to assess mortality differences between groups, which were defined as follows: baseline group—patients not on H2RAs who were not ventilated; group 1—patients not on H2RAs who were ventilated; group 2—patients on H2RAs who were not ventilated; and group 3—patients on H2RAs who were ventilated. Mortality of these subgroups were assessed using univariate and multivariable logistic regression controlling for the same covariates as above. We compared daily median PF ratio, BUN/Cr ratio, BUN, creatinine, AST, and ALT between patients who used H2RA and those who did not using Wilcoxon signed rank test. Median confidence intervals were calculated based on David Olive’s method [30]. Proportions of patients in the H2RA and No H2RA group categorized as no ARDS, mild ARDS, moderate ARDS, and severe ARDS were calculated on days 1 through 7 from ICU admission to further assess lung function. Mann-Kendall’s test was used to assess linear trends in the proportion of patients in each group among the four categories of lung function (where positive tau values mean an increasing linear trend). Data cleaning and analysis utilized RStudio version 1.4.1106 (RStudio Team, 2020) and Jamovi 2 [31–37]. All analyses were performed as two-sided with a 0.05 level of significance.

## Results

### Baseline characteristics

We included 35,010 patients who met sepsis 3 criteria and were admitted to the ICU at some point in their hospital stay from the years 2008 to 2019. We excluded 4419 patients who were either mechanically ventilated prior to ICU admission or received H2RAs after 1 day into their ICU stay. Of these, 12,908 were in the H2RA group (42.2%) and 17,683 (57.8%) were in the No H2RA group. Table 1 summarizes baseline characteristics of patients included in this study. The median age of the No H2RA group was significantly older than the H2RA group (69 vs 66 years, *p* < 0.001). Males were overrepresented in the overall cohort and were significantly less in the no H2RA group compared to the H2RA group (55.66% vs 60.65%, *p* < 0.001). Comorbidities investigated showed significant differences in all categories, effect sizes rarely were greater than 5%. Notable differences included cerebrovascular disease [no H2RA vs. H2RA] (10.98% vs 17.48%, *p* < 0.01), chronic pulmonary disease (30.16% vs 25.23%, *p* < 0.01), congestive heart failure (36.76% vs 28.12%, *p* < 0.01), malignant cancer (16.23% vs 11.07%, *p* < 0.01), mild liver disease (19.32% vs 10.51%, *p* < 0.01), renal disease (30.96% vs 19.62%, *p* < 0.01), and severe liver disease (11.34% vs 4.03%, *p* < 0.01). Small but significant differences were found for median Charlson comorbidity index (6 vs 5, *p* < 0.01). Small but significant differences in median were also found in three of the four risk scores including APS III (51 vs 48, *p* < 0.01), OASIS (33 vs 35, *p* < 0.01), and SOFA score (5 vs 6, *p* < 0.01). There was no difference in the median SAPS II score.

**Table 1:** Summary of baseline patient characteristics

	No H2RA (***n*** = 17,683) (57.8%)	H2RA (***n*** = 12,908) (42.2%)	***p*** value
Age, [median, years (IQR)]	69 (58–80)	66 (56–76)	< 0.01
Males	9842 (55.7%)	7829 (60.7%)	< 0.01
**Race**			< 0.01
American Indian/Alaska Native	50 (0.3%)	19 (0.2%)	
Asian	508 (2.9%)	374 (2.9%)	
Black/African American	2062 (11.7%)	1078 (8.4%)	
Hispanic/Latino	640 (3.6%)	498 (3.9%)	
Other	754 (4.3%)	657 (5.1%)	
Unknown	1405 (7.9%)	1724 (13.4%)	
White	12,264 (69.4%)	8558 (66.3%)	
**Comorbid conditions**			
AIDS	197 (1.1%)	82 (0.6%)	< 0.01
Cerebrovascular disease	1942 (11.0%)	2256 (17.5%)	< 0.01
Chronic pulmonary disease	5334 (30.2%)	3257 (25.2%)	< 0.01
Congestive heart failure	6500 (36.8%)	3630 (28.1%)	< 0.01
Dementia	1072 (6.1%)	373 (2.9%)	< 0.01
Diabetes, chronic complications	2291 (13.0%)	1153 (8.9%)	< 0.01
Diabetes no chronic complications	4629 (26.2%)	3042 (23.6%)	< 0.01
Malignant cancer	2870 (16.2%)	1429 (11.1%)	< 0.01
Metastatic solid tumor	1334 (7.5%)	610 (4.7%)	< 0.01
Mild liver disease	3416 (19.3%)	1357 (10.5%)	< 0.01
Myocardial infarction	3147 (17.8%)	2269 (17.6%)	0.62
Paraplegia	653 (3.7%)	817 (6.3%)	< 0.01
Peptic ulcer disease	850 (4.8%)	204 (1.9% )	< 0.01
Peripheral vascular disease	2184 (12.4%)	1748 (13.5%)	< 0.01
Renal disease	5474 (31.0%)	2533 (19.6%)	< 0.01
Rheumatic disease	764 (4.3%)	405 (3.1%)	< 0.01
Severe liver disease	2006 (11.3%)	520 (4.0%)	< 0.01
Charlson comorbidity index [median (IQR)]	6 (5–8)	5 (4–7)	< 0.01
APS III Score [median (IQR)]	51 (39–68)	48 (34–70)	< 0.01
SAPS II Score [median (IQR)]	38 (33–48)	38 (30–47)	< 0.01
OASIS Score [median (IQR)]	33 (27–39)	35 (29–41)	< 0.01
First Day SOFA Score [median (IQR)]	5 (4–8)	6 (4–9)	< 0.01

### All-cause mortality

**Fig. 1:**
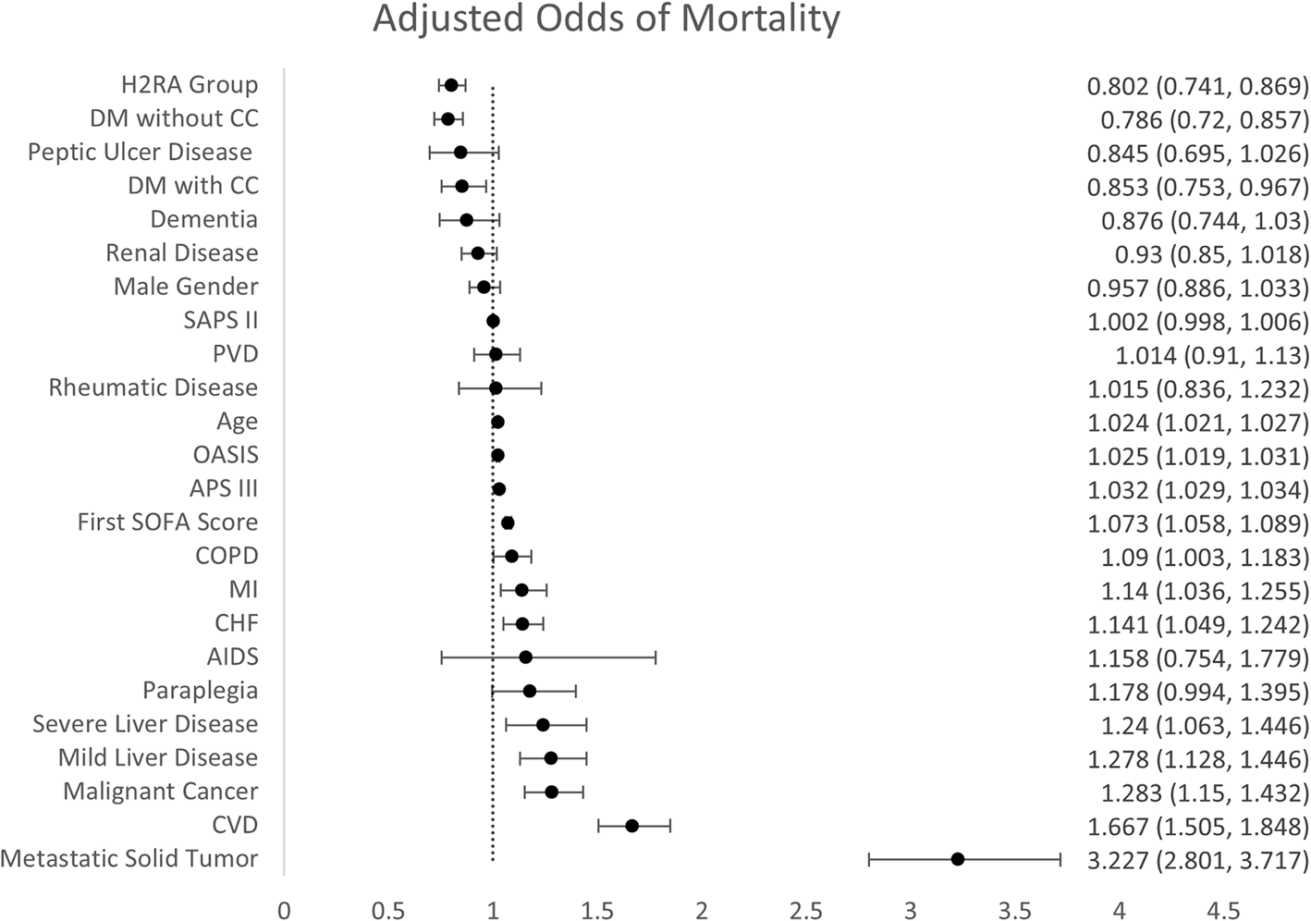
Predictors of 28-day mortality among patients who stayed in the ICU for sepsis management

The 28-day in hospital mortality rate was significantly lower among patients who used H2RAs prior to their ICU stay (12.6% vs 15.1%, *p* < 0.001). A multivariable logistic regression demonstrated that patients in the H2RA group had an adjusted odds ratio (OR) of 0.802 (0.741–0.869, *p* < 0.001) for 28-day in hospital mortality. Figure 1 shows the relative strengths of the predictors of 28-day mortality in our model.

### Length of stay

H2RA use was associated with a significantly longer median length of ICU stay (3.2 days vs 2.4 days, *p* < 0.001). Multivariable linear regression analysis demonstrated a significant association between receiving H2RAs and increased ICU length of stay (LOS) (difference in days: 1.569, standard estimate 0.23, *p* < 0.001). Other significant predictors of increased ICU length of stay included APS III (difference in days 0.113, standard estimate: 0.31, *p* < 0.001), SAPS II (difference in days − 0.14, standard estimate − 0.31, *p* < 0.001), dementia (difference in days − 1.988, standard estimate − 0.24, *p* < 0.001), metastatic solid tumor (difference in days − 1.472, standard estimate − 0.23, *p* < 0.001), oasis (difference in days 0.158, standard estimate 0.21, *p* < 0.001), cerebrovascular disease (difference in days 1.39, standard estimate 0.19, *p* < 0.001), severe liver disease (difference in days − 1.697, standard estimate − 0.19, *p* < 0.001), paraplegia (difference in days 1.042, standard estimate 0.18, *p* < 0.001), Charlson comorbidity index (difference in days 0.427, standard estimate 0.17, *p* < 0.001), first SOFA score (difference in days 0.353, standard estimate 0.15, *p* < 0.001), mild liver disease (difference in days − 0.887, standard estimate − 0.14, *p* < 0.001), diabetes with complicating condition (difference in days − 1.082, standard estimate − 0.13, *p* < 0.001), renal disease (difference in days − 1.222, standard estimate − 0.13, *p* < 0.001), rheumatic disease (difference in days − 0.889, standard estimate − 0.12, *p* < 0.001), diabetes without complicating condition (difference in days − 0.768, standard estimate − 0.09, *p* < 0.001), myocardial infarct (difference in days − 0.573, standard estimate − 0.08, *p* < 0.001), peptic ulcer disease (difference in days 0.135, standard estimate 0.08, *p* < 0.01), age (difference in days − 0.027, standard estimate − 0.06, *p* < 0.001).

### Need for invasive mechanical ventilation

**Fig. 2:**
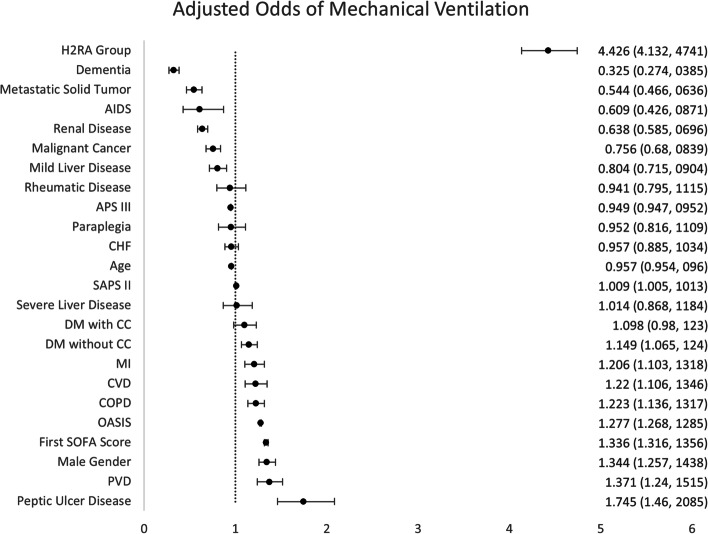
Predictors of need for invasive mechanical ventilation among patients who stayed in the ICU for sepsis management

Significantly more patients in the H2RA group required invasive mechanical ventilation than the No H2RA group (62.15% vs. 31.81%, *p* < 0.01). Multivariable logistic regression demonstrated that patients in the H2RA group had an adjusted odd ratio of 4.426 (95% CI [4.132 – 4.741], *p* < 0.001) for needing mechanical ventilation (Fig. 2).

### Association between H2RA use and IMV on mortality and length of stay outcomes

To assess differences in mortality based on a combination of H2RA use and the need for IMV, we identified four sub-groups (defined in methods above) and compared mortality outcomes between them. Mortality rate differed significantly between the groups (Table 2). The baseline group was not on H2RAs and did not receive IMV. Mortality rate in this group was 10.2%. Patients in group 2 who received H2RAs and did not require IMV, had the lowest mortality rate of 6.7%. Mortality rate was highest, 25.4% among those who were on IMV but did not receive H2RA (group 2). Multivariable logistic regression demonstrated significant differences between group 2 vs the baseline group (OR 0.79 95% CI [0.686–0.911], *p* = 0.001) and group 1 vs the baseline group (OR 1.419 95% CI [1.265–1.591], *p* < 0.001) (Table 3). No significant difference was found between group 3 and the baseline group (OR 1.042 95% CI [0.93–1.168], *p =* 0.474). Among groups of patients with IMV, i.e., groups 1 and 3, there was a significant increased odds of mortality in group 1, who did not receive H2RAs, compared to group 3 (OR 1.36 95% CI [1.23–1.50], *p* < 0.001).

**Table 2:** Subgroup analysis of the relationship between H2RA use and mechanical ventilation status and mortality and length of stay outcomes

	Baseline group No H2RA, no IMV (*N* = 12,058)	Group 1 No H2RA, IMV (*N* = 5625)	Group 2 H2RA, no IMV (*N* =4499)	Group 3 H2RA, IMV (*N* = 8409)	Total (*N* = 30,591)	*p* value
**Mortality**						< 0.001
Survived	10,823 (89.8%)	4198 (74.6%)	4196 (93.3%)	7089 (84.3%)	26,306 (86.0%)	
Died	1235 (10.2%)	1427 (25.4%)	303 (6.7%)	1320 (15.7%)	4285 (14.0%)	
Median ICU LOS (IQR)	1.9 (1.1–3.2)	2 (1.2–3.2)	4.6 (2.4–8.9)	4.7 (2.2–10)		
Mean ICU LOS (SD)	2.6 (2.4)	6.9 (7.2)	2.8 (2.7)	7.8 (9.4)		< 0.001

Definitions: *Baseline group* patients not on H2RAs who were not ventilated, *Group 1* patients not on H2RAs who were ventilated, *Group 2* patients on H2RAs who were not ventilated and *Group 3* patients on H2RAs who were ventilated

**Table 3:** Multivariable logistic regression of 28-day mortality in subgroup analysis of mechanical ventilation and H2RA groups (note: same covariates were used in these models as the mortality analysis)

Predictor	Odds ratio	*p* value	95% confidence interval
Reference group: baseline			
Group 1	1.438	< .001	1.282–1.613
Group 2	0.808	0.003	0.701–0.932
Group 3	1.074	0.223	0.957–1.206

In subgroup analysis of LOS, we found that patients who received IMV had significantly longer mean LOS (H2RA group 7.8 days; no H2RA group 6.9 days) while the patients who were not mechanically ventilated had significantly lower mean LOS (H2RA group 2.8 days; no H2RA group 2.6 days) (Table 2). Furthermore, between just the ventilated patients, the mean LOS was statistically significant (*p* < 0.001) with H2RA patients on mechanical ventilation having longer length of stay.

### Association between H2RA use and organ dysfunction

**Fig. 3:**
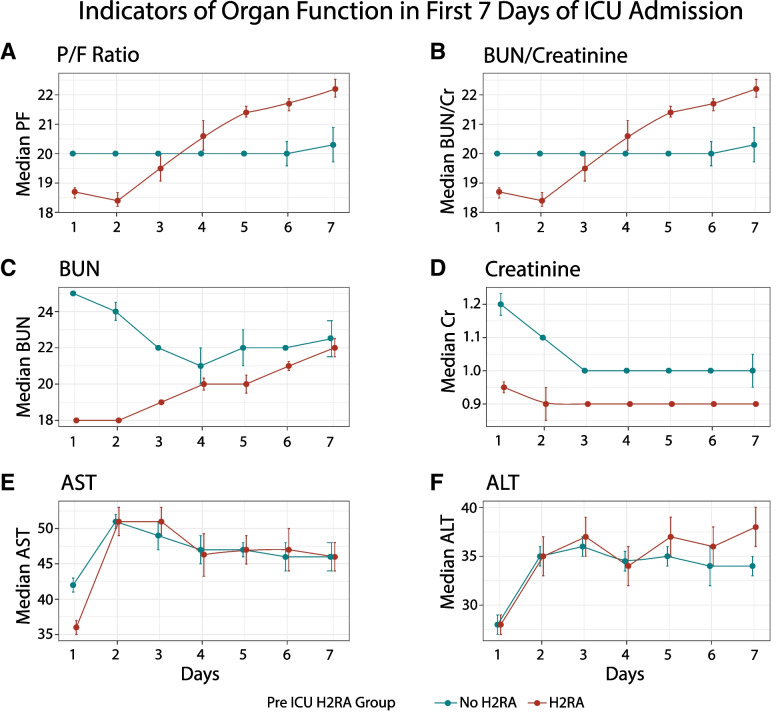
Daily trend in lung, kidney, and liver function over the first seven days of ICU stay

**Fig. 4:**
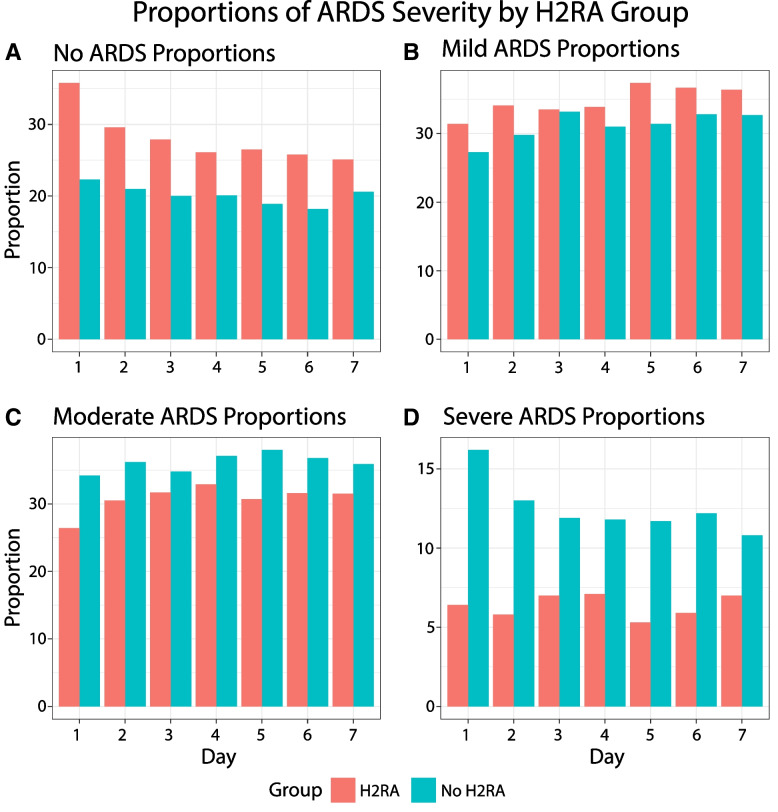
Daily proportions of patients in each ARDS severity group from days 1 to 7 of ICU admission

Patients receiving H2RAs had significantly higher median P/F ratios on days 1 through 7 of ICU stay (*p* < 0.05) (Fig. 3A). Overall, H2RA use is associated with better lung function on most days as indicated by higher PF ratios. In the H2RA group compared to the no H2RA group, significantly higher proportion (*p* < 0.05) of patients were in the no ARDS and mild ARDS groups on all days except for day 3 (Fig. 4). Median BUN/Cr ratios were significantly lower (*p* < 0.05) on days 1–3 for patients in the H2RA group while these were significantly higher on days 4–7 (Fig. 3). BUN medians were significantly lower in the group receiving H2RAs on most days (Fig. 3). Serum creatinine medians were significantly lower on all days following ICU admission in the H2RA group (Fig. 3). Median AST and ALT were generally not significantly different in the H2RA group versus the no H2RA group.

## Discussion

H2RAs are widely used for the management of acid-peptic disease, including gastroesophageal reflux disease, gastric and duodenal ulcers and for SUP in critically ill patients. In this retrospective cohort study of ICU patients with sepsis, we assessed the association between the use of H2RAs and sepsis outcomes based on the demonstrated role of histamine antagonism in attenuating sepsis-related organ dysfunction in mouse models.

Our analysis shows that H2RA administration is associated with decreased mortality for non-ventilated and ventilated patients with sepsis. Notably, decreased mortality was observed despite a slightly higher median first day SOFA score among patients who received an H2RA as compared to the non-H2RA group. Patients receiving an H2RA also had higher average P/F ratios and were less likely to develop moderate or severe ARDS as compared to those who were not treated with an H2RA. Interestingly, despite the mortality benefit, H2RA use was associated with an increased rate of invasive mechanical ventilation, and longer ICU LOS. The increased risk of invasive mechanical ventilation among patients receiving an H2RA most likely reflects correlation rather than causation: H2RAs are routinely administered prophylactically to patients on invasive mechanical ventilation to reduce the risk of gastrointestinal hemorrhage. In our analysis, both ventilated and non-ventilated patients receiving an H2RA had significantly reduced odds of mortality as compared to those who did not receive an H2RA.

Prolonged ICU LOS among patients receiving an H2RA may also reflect correlation rather than causation. H2RAs were administered more frequently to ventilated patients as compared to non-ventilated patients. Increased ventilation requirements typically indicate more severe disease and thus it is expected that ventilated patients will require a longer LOS as compared to non-ventilated patients, irrespective of H2RA administration. Notably, however, ventilated patients who received an H2RA required approximately one day longer LOS as compared to ventilated patients who did not receive an H2RA. Similarly, non-ventilated patients who received an H2RA required approximately 2 h longer LOS as compared to non-ventilated patients who did not receive an H2RA. The underlying cause of prolonged LOS among patients treated with an H2RA requires further investigation.

The decreased mortality observed in patients taking H2RAs in mechanically ventilated and non-ventilated patients despite prolonged ICU length of stay is somewhat paradoxical and warrants careful examination. We hypothesize that H2RAs may improve pulmonary function in the setting of sepsis via a dual mechanism involving a reduction in alveolar wall edema and modulation of airway and vascular smooth muscle inflammation. Previous studies have shown that histamine 2 receptors are expressed on mast cells; antagonism of mast cell histamine 2 receptors may decrease degranulation and attenuate local alveolar wall edema [38]. In addition, antagonism of histamine 2 receptors expressed by airway and vascular smooth muscles can reduce pulmonary vascular resistance and thus improve hemodynamic and functional status [39]. In our analysis, we provide data suggest improved pulmonary function among patients receiving H2RAs, as evidenced by higher average P/F ratios and decreased rates of moderate or severe ARDS.

H2RAs demonstrate a wide range of extraintestinal effects. They have been shown to reduce ventricular remodeling by interrupting histamine-mediated myocardial remodeling. The Multi-Ethnic Study of Atherosclerosis (MESA) right ventricle study established that H2RA use was associated with lower right ventricular mass and end-diastolic volume among individuals with risk factors for cardiovascular disease [40, 41]. Other purported extraintestinal benefits of H2RAs are far-reaching and include reduction of bladder pain from interstitial cystitis, improvement of cell-mediated immunity, and reduction of symptoms related to erythropoietic protoporphyria [42–44]. Furthermore, recent studies have demonstrated a potential role for H2RAs as an adjunctive treatment for COVID-19 [45, 46]. To the best of our knowledge, this is the first large-scale study to assess the effect of H2RAs on mortality in the setting of sepsis.

The study is strengthened by a large sample size with many data elements per subject. The MIMIC-IV database provides risk scores, disease severity indices, and extensive comorbidity information for each subject, which allowed us to control for potential confounding variables. This study also considers only patients with sepsis, whereas other studies on GI prophylactic medications have included all critically ill patients with a range of possible etiologies for their admissions. However, there are several limitations of our study inherent to its design. First, the analysis was retrospective and thus causation could not be assessed. Second, H2RAs were considered as a class thus we are unable determine if the relationships observed are a class effect. Further, we were unable to include specific doses of agents in our analysis; therefore, dose effects could not be assessed. Third, the specific cause of death is not included in the MIMIC-IV database. We therefore report crude mortality rates. The mechanisms underlying the mortality benefit associated with H2RAs could conceivably be more clearly defined if cause of death was established. Fourth, the no-H2RA group likely included a heterogenous group of patients who could ostensibly be receiving other GI prophylaxis, or no GI prophylaxis at all. Lastly, the data utilized in this study was limited to a single center, which may limit the generalizability of our findings.

In ICU patients with sepsis, the use of H2RAs is associated with significantly lower adjusted odds and incidence of all-cause mortality irrespective of the initial severity of sepsis and invasive mechanical ventilation status. The underlying mechanism for this observation is unclear but may be due to amelioration of histamine-related dysfunction at the capillary beds with resultant improvement in tissue perfusion. We speculate that H2RAs restore the integrity of the vascular membrane, reduce alveolar wall edema, and mitigate airway and vascular smooth muscle inflammation. H2RAs are routinely used in stress ulcer prophylaxis in mechanically ventilated patient. Our findings suggest that H2RAs may provide extraintestinal benefits in ICU patients with sepsis; although PPIs may be preferred among patients with a high risk of gastrointestinal hemorrhage, we propose that H2RAs should be considered for patients with a high risk of pneumonia and other cardiopulmonary complications Further research is warranted to clearly define the mechanisms underlying histamine-mediated end organ damage and establish potential applications for H2RAs among patients with sepsis.

## Data Availability

The datasets analyzed during the current study are publicly available in the MIMIC-IV database (https://physionet.org/content/mimiciv/1.0/).

## Abbreviations

AIDS: Acquired immunodeficiency syndrome
ALT: Alanine Aminotransferase
APS III: Acute Physiology Score III
ARDS: Acute respiratory distress syndrome
AST: Aspartate aminotransferase
MIMIC-IV: Medical Information Mart for Intensive Care IV
BUN: Blood urea nitrogen
CIB: Clinically important bleeding
H2RA: Histamine-2 receptor antagonists
ICU: Intensive care unit
IMV: Invasive mechanical ventilation
LOS: Length of stay
OASIS: Outcome Assessment Information Set
SAPS II: Simplified Acute Physiology Score II
SOFA: Sequential Organ Failure Assessment
SUP: Stress ulcer prophylaxis
